# Predictors of adverse drug reaction-related hospitalisation in Southwest Ethiopia: A prospective cross-sectional study

**DOI:** 10.1371/journal.pone.0186631

**Published:** 2017-10-16

**Authors:** Mulugeta Tarekegn Angamo, Colin Michael Curtain, Leanne Chalmers, Daniel Yilma, Luke Bereznicki

**Affiliations:** 1 Division of Pharmacy, School of Medicine, University of Tasmania, Hobart, Tasmania, Australia; 2 Department of Internal Medicine, School of Medicine, Faculty of Medical Sciences, Jimma University, Jimma, Ethiopia; University of Oxford, UNITED KINGDOM

## Abstract

**Background:**

Adverse drug reactions (ADRs) are important causes of morbidity and mortality in the healthcare system; however, there are no studies reporting on the magnitude and risk factors associated with ADR-related hospitalisation in Ethiopia.

**Objectives:**

To characterise the reaction types and the drugs implicated in admission to Jimma University Specialized Hospital, Southwest Ethiopia, and to identify risk factors associated with ADR-related hospitalisation.

**Methods:**

A prospective cross-sectional study was conducted from May 2015 to August 2016 among consenting patients aged ≥18 years consecutively admitted to medical wards taking at least one medication prior to admission. ADR-related hospitalisations were determined through expert review of medical records, laboratory tests, patient interviews and physical observation. ADR causality was assessed by the Naranjo algorithm followed by consensus review with internal medicine specialist. ADR preventability was assessed using Schumock and Thornton’s criteria. Only definite and probable ADRs that provoked hospitalisation were considered. Binary logistic regression was used to identify independent predictors of ADR-related hospitalisation.

**Results:**

Of 1,001 patients, 103 (10.3%) had ADR-related admissions. Common ADRs responsible for hospitalisation were hepatotoxicity (35, 29.4%) and acute kidney injury (27, 22.7%). The drug classes most frequently implicated were antitubercular agents (45, 25.0%) followed by antivirals (22, 12.2%) and diuretics (19, 10.6%). Independent predictors of ADR-related hospitalisation were body mass index (BMI) <18.5 kg/m^2^ (adjusted odd ratio [AOR] = 1.69; 95% confidence interval [CI] = 1.10–2.62; p = 0.047), pre-existing renal disease (AOR = 2.84; 95%CI = 1.38–5.85, p = 0.004), pre-existing liver disease (AOR = 2.61; 95%CI = 1.38–4.96; p = 0.003), number of comorbidities ≥4 (AOR = 2.09; 95%CI = 1.27–3.44; p = 0.004), number of drugs ≥6 (AOR = 2.02; 95%CI = 1.26–3.25; p = 0.004) and history of previous ADRs (AOR = 24.27; 95%CI = 11.29–52.17; p<0.001). Most ADRs (106, 89.1%) were preventable.

**Conclusions:**

ADRs were a common cause of hospitalisation. The majority of ADRs were preventable, highlighting the need for monitoring and review of patients with lower BMI, ADR history, renal and liver diseases, multiple comorbidities and medications. ADR predictors should be integrated into clinical pathways and pharmacovigilance systems.

## Introduction

Adverse drug reactions (ADRs) are one of the leading causes of morbidity and mortality in the healthcare system [1]. Globally, studies have reported that the overall prevalence of ADR-related hospitalisation varies from 0.2% [2] to 54.5% [3]. A recent review of 43 observational studies identified a comparable prevalence of ADR-related hospitalisation in developed and developing countries [1]. However, most of the studies included in this review were conducted in developed countries, where the disease characteristics and prevalence, access to medicines, drug use patterns and management systems differ markedly from those in developing countries [4]. With respect to potential risk factors for ADRs, developing countries differ from developed countries in several important areas. These include greater proportions of patients taking antituberculosis (anti-TB) and antiretroviral therapy (ART) [5], a high prevalence of anaemia and malnutrition [6], widespread use of traditional remedies [7], a higher incidence of concomitant anti-TB drugs and ART with overlapping adverse effects [6], and increasing rates of concomitant infectious and non-communicable diseases demanding multiple medications with potential interactions [8–12]. There are also higher frequencies of genetic variants conferring increased risk for ADRs for commonly used drugs treating cancer, human immunodeficiency virus (HIV)/acquired immunodeficiency syndrome (AIDS) and tuberculosis (TB) in persons of African descent [13].

In Ethiopia, a developing country, the risk of ADR-related hospitalisation is a health concern due to an increasing number of patients eligible for anti-TB and ART, due to decentralisation and scale-up of the HIV/AIDS care programme [14], and concomitant drug management of HIV/TB co-infected patients [14]. According to a 2014 World Health Organization (WHO) report, the prevalence of all forms of TB was 211 per 100,000 of the population, of whom 13% of patients were HIV co-infected [15]. The prevalence of HIV among the adult population in 2015 was estimated to be 1.0% with national ART coverage of 52.0% [16]. There is growing attention to chronic diseases management with multiple medications [11, 17]. The use of new and complex therapies for chronic diseases has increased due to the establishment of community care programmes [18] and strengthening of health systems for chronic care at the level of local health centres, primary, general, and specialised hospitals [18]. The provision of primary health care services by health extension workers (health professionals working at the lowest health care level targeting preventive and curative health services at the household level) [19] and the expansion of community pharmacies and drug stores at the community level has remarkably increased medication access and use in the community, although medication regulation is poor [20]. There are also higher rates of drug-related problems [21] and irrational use of medicines [22] among patients with chronic illnesses in ambulatory care clinics that could lead to drug-related harm.

Studies have identified several factors contributing to ADR-related hospitalisations including older age [23, 24], female gender [25, 26], increased number of co-morbidities [27, 28], increased number of medications [29, 30], renal diseases [31, 32], liver diseases [33], heart failure [28], higher Charlson Comorbidity Index (CCI) [34], presence of chronic illnesses [35, 36], and history of previous ADRs [37, 38]. HIV/AIDS patients taking ART have been identified as a risk factor for ADR-related hospitalisations only in developing countries [8, 39]. Drugs commonly reported as contributing to ADRs include anticoagulants [24, 40], non-steroidal anti-inflammatory drugs (NSAIDs) [41], and angiotensin-converting enzyme inhibitors [41]. However, studies focussing on risk factors for ADR-related hospitalisation in developing countries are very limited in number [1]. Therefore, identification and reporting of factors contributing to ADR-related hospitalisation for community-based patients is crucial to develop preventive strategies to decrease the burden in the developing world [9, 10].

To our knowledge, there are no studies reporting on the prevalence and risk factors associated with ADR-related hospitalisation in Ethiopian patients. Thus, the main aim of this study was to characterise the reaction types and the drugs implicated in admission to Jimma University Specialized Hospital (JUSH), Southwest Ethiopia, and to identify risk factors associated with ADR-related hospitalisation.

## Methods

### Study setting, design and population

This study was conducted at the JUSH, which is the major public hospital in southwest Ethiopia with a catchment population of about 15 million people [42].

A prospective cross-sectional study was conducted from May 2015 to August 2016. Consenting patients with complete medical records aged ≥18 years and taking at least one medication prior to admission to medical wards were included in the study. Patients were excluded if they were unwilling to participate, unable to be interviewed due to health or other reasons, not taking at least one medication prior to admission and had incomplete medical and medication records.

### Data collection

One of the authors (MTA) interviewed consecutively admitted patients as soon as practical for socio-demographic information, social drug use, medical history, drug allergies, use of over-the-counter and herbal medicines (S1 Table). Patients’ medical records were reviewed for admission diagnosis, ADR history, and clinical data within 48 hours of admission. Medication exposure in the month preceding hospitalisation was obtained through review of patients’ medical records and /or interview with the patient or family members. Common laboratory tests were evaluated within 48 hours of admission for each patient case including: renal function (serum creatinine mg/dL, blood urea nitrogen mg/dL and estimated glomerular filtration rate (eGFR) mL/min/1.73m^2^), liver function (alanine transaminase IU/L, aspartate transaminase IU/L, alkaline phosphatase IU/L and total bilirubin mg/dL) and complete blood count (white blood cell count cells/mm^3^, red blood cell count cells/mm^3^, haemoglobin g/dL, haematocrit %, and platelet count cells/mm^3^). Parameters describing nutritional and metabolic status (serum albumin g/dL, total triglyceride mg/dL, low-density lipoprotein mg/dL, high-density lipoprotein mg/dL, glucose mg/dL), serum electrolytes (serum calcium mmol/L, potassium mmol/L and sodium mmol/L) and coagulation status (prothrombin time) were recorded. Vital statistics such as body temperature, blood pressure, respiratory rate and pulse rate were recorded at admission. Other diagnostic data, such as echocardiography, ultrasound, electrocardiogram and viral markers and urinalysis results, were also evaluated.

### Definitions of terms and variables used

The WHO definition of an ADR was used in this study: “any response to a drug which is noxious and unintended, and which occurs at doses normally used in man for prophylaxis, diagnosis, or therapy of disease, or for the modification of physiological function” [43]. This definition excludes treatment failure, drug abuse, intentional drug overdose and accidental or self-poisoning. Alcohol consumption was recorded as number of standard drinks per day. Khat chewing, which was of interest as it contributes to elevated blood pressure [44], was defined as regular chewer if a patient chewed khat at least four times per week. Drugs were classified using the WHO Anatomical Therapeutic Chemical Classification (ATC) System [45] and diagnoses were coded according to International Classification of Primary Care 2^nd^ edition [46]. Calculation of the number of medications was based on the number of active ingredients in single and combination products [47]. A cut off point for polypharmacy was ≥6 medications [48]. A cut off point for comorbidities was ≥4 diseases based on a similar study [39]. Adult nutritional status was assessed using the body mass index (BMI) [49] and classified as <18.5 kg/m^2^ and ≥ 18.5 kg/m^2^ for analysis purpose. Patients were considered to have pre-existing chronic kidney disease if the eGFR was <60 mL/minute/1.73m^2^ and they had documented abnormal renal ultrasound (abnormal renal echogenicity or kidney size or presence of cysts) for at least 3 months prior to admission [50]. Drug-induced acute kidney injury was suspected among patients with baseline renal insufficiency (eGFR < 60 mL/minute/1.73m^2^), volume depletion and multiple exposures to nephrotoxic agents prior to admission, as long as other potential causes were excluded. Chronic liver disease was considered pre-existing if liver diseases (such as cirrhosis, chronic viral hepatitis) or liver dysfunction or liver injury were documented by the treating physician prior to admission [33]. Drug-induced hepatotoxicity was suspected when aspartate aminotransferase (AST) or alanine aminotransferase (ALT) levels were 3 or more times the upper normal limit (UNL) or total bilirubin was 2 or more times UNL [51], as long as other potential causes were excluded. The response to rechallenge or re-exposure to some drugs, such as anti-TB drugs, were implemented based on the WHO guidelines for the treatment of TB [52]. HIV/AIDS and TB patients were those patients diagnosed with HIV/AIDS and TB, respectively, and taking ART and anti-TB drugs prior to the current admission. All comorbidities were defined as present if documented in the medical records. A patient who had studied at least to a primary school level and was able to read and write in the local language(s) was considered as educated, whereas other patients were considered uneducated. According to the Ethiopian context, cities and small towns were considered as urban areas while rural villages or other similar clusters were considered as rural areas.

### Identifying ADRs as a cause for hospitalisation

The primary researcher (MTA) evaluated all patients admitted to medical wards during the study period to assess if the admission had been caused by an ADR. The identification of whether one or more drugs led to the hospitalisation was based on a review of medical records, evaluation of laboratory tests, interview with patients or family members about medication usage and physical observation. An ADR was suspected if there was a relationship between the time of drug administration and the onset and course of the adverse reaction, while excluding other potential causes. The known adverse reaction profile of each drug was evaluated based on Ethiopian National Drug Formulary (2014), British National Formulary [53] and Up-To-Date 19.3 [54]. Confirmation of the causal relationship of an ADR to the suspected medication was performed using the Naranjo ADR assessment scale [55]. Applying the Naranjo algorithm, ADRs were classified as definite (9–12 points), probable (5–8 points), possible (1–4 points), or doubtful (0 points). The senior supervising internal medicine specialist (DY) independently and blindly reviewed all cases of suspected ADRs and cases without suspected ADRs for the presence of ADRs and to confirm the Naranjo rating using a similar approach to other studies [56, 57]. The primary researcher (MTA) and the senior supervising internal medicine specialist met to reach a consensus decision on the presence of an ADR-related admission and excluded possible and doubtful cases. When consensus was not reached on the causality assessment, an additional clinical pharmacy specialist’s opinion was sought for majority decision. Only definite and probable ADRs that provoked hospitalisation were considered. ADRs were assessed for preventability using Schumock and Thornton criteria [58] through the same approach. ADRs were classified as type A (dose dependent, augmented pharmacological and predictable reactions) and type B (bizarre, dose independent and non-predictable reactions) according to the Rawlins and Thompson classification method [59]. ADRs observed during the hospital stay were excluded.

### Data analysis and interpretation

Data was recorded into an Access database (2016, Microsoft, Redmond, Washington) and analysed using the IBM Statistical Package for the Social Science (IBM SPSS version 23.0 Inc., Chicago, Illinois). Frequency of adverse reaction types and drugs implicated were determined. The chi-square test or Fisher’s exact test were used to compare categorical data between ADR- and non-ADR patients. For non-normally distributed variables, comparisons were undertaken with Mann-Whitney tests and the results were presented as medians and interquartile ranges (IQR). For normally distributed continuous variables, comparisons were performed using Student’s t-tests with results presented as means and standard deviations (SD).

Independent variables were assessed for multicollinearity and association to rule out correlation between two or more independent variables using variance inflation factor. Independent variables with p<0.25 in univariate analyses were entered into a multivariable binary logistic regression model to determine independent predictors of ADR-related hospitalisation. The performance of the ADR-related admission risk prediction model was assessed using the area under the receiver operator curve (AUROC), which assesses the ability of independent risk score to predict ADR-related admissions. The ROC curve was prepared using R (2015, R Foundation for Statistical Computing, Vienna) [60]. A p value of <0.05 was considered statistically significant in all analyses.

### Ethics

The study was approved by the Tasmanian Health and Medical Human Research Ethics Committee (reference number H0014718) and the Jimma University Institutional Review Board (reference number RPGC/58/2015). We received permission from Jimma University Specialised Hospital management to conduct the research. Written informed consent was obtained from all individual participants included in the study.

## Results

### General information

**Fig 1:**
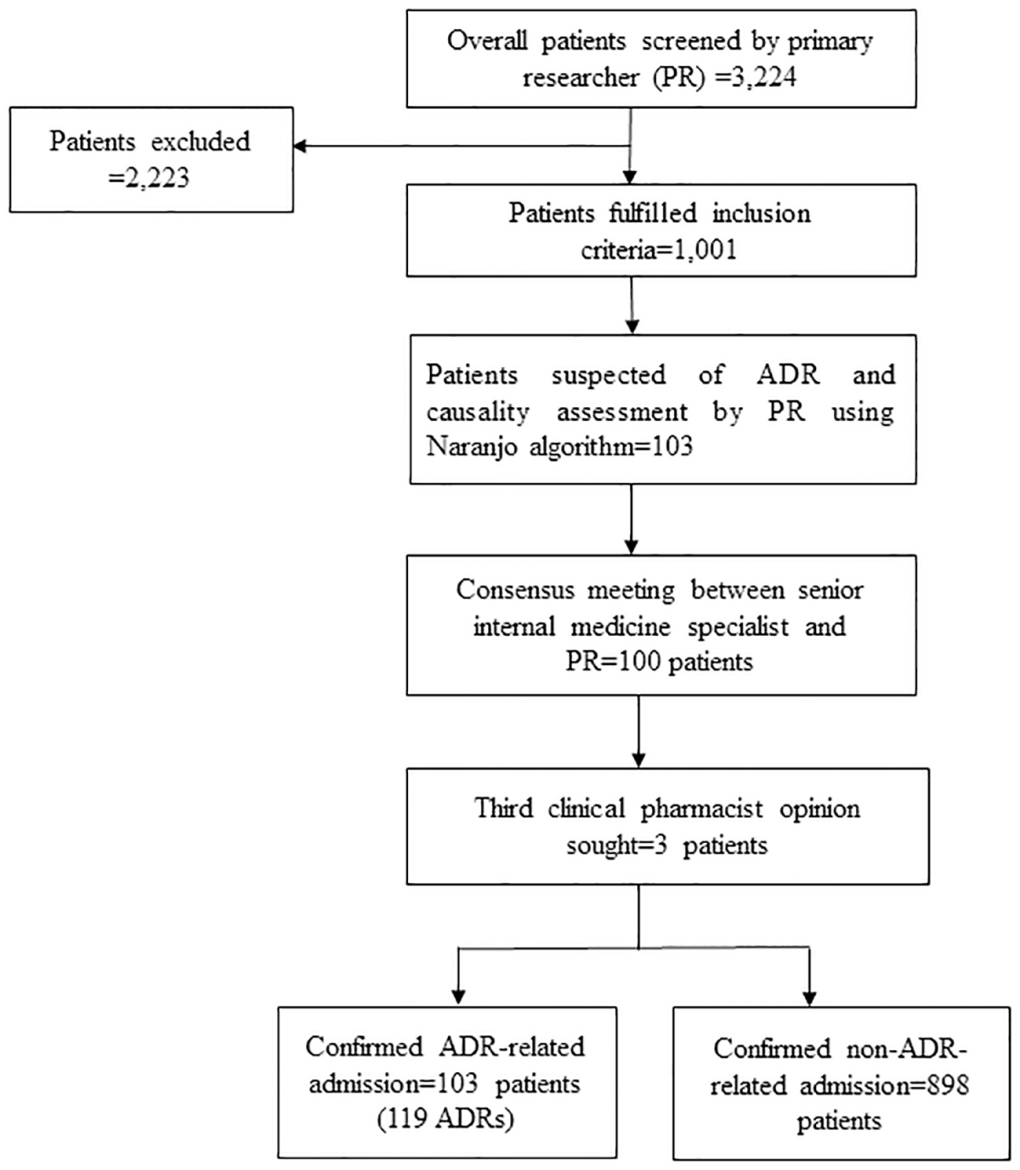
The diagram outlining patient screening and identification of ADR-related admissions.

A total of 3,224 patients were screened; 2,223 patients were excluded due to age <18 years (255), not taking at least one medication prior to admission (576), incomplete medical and medication records (70), unwillingness to participate (463) and inability to be interviewed as a result of health or other reasons (859). Of the 1,001 patients who met the inclusion criteria, 103 (10.3%) were deemed to have experienced an ADR-related admission (Fig 1).

One hundred and nineteen ADRs were identified among the 103 patients, equating to 1.2 ADRs per patient. Of these, 26.1% were definite and 73.9% were probable. Most ADRs (106, 89.1%) were considered preventable. Ninety-nine (83.2%) of the ADRs were considered pharmacologically predictable (type-A reactions) (Table 1).

**Table 1:** Causality, type and preventability of ADRs causing hospitalisation (N = 119).

ADR classifications	n (%)
Causality of ADRs	
Definite	31(26.1)
Probable	88 (73.9)
Preventability scale	
Not preventable	13 (10.9)
Definitely preventable	19 (16.0)
Probably preventable	87 (73.1)
Rawlins classification of reaction	
Type A (pharmacologically predictable)	99 (83.2)
Type B (pharmacologically non-predictable)	20 (16.8)

### ADR characteristics and drugs implicated in ADRs

The most common ADRs responsible for hospital admissions were hepatotoxicity (35, 29.4%) followed by acute kidney injury (severely declined glomerular filtration rate and hypovolemia) (27, 22.7%), skin reactions (8, 6.7%), hypokalaemia (7, 5.9%) and gastrointestinal bleeding or gastritis (7, 5.9%). Commonly implicated drugs in hepatotoxicity were isoniazid (21, 11.7%) followed by pyrazinamide (18, 10.0%), efavirenz (5, 2.8%) and atorvastatin (5, 2.8%). Commonly implicated drugs in acute kidney injury were tenofovir (9, 5.0%), furosemide (7, 3.9%), and enalapril (7, 3.9%). The drugs most commonly suspected of causing skin reactions were sulfamethoxazole (2, 1.1%), trimethoprim (2, 1.1%) and rifampicin (2, 1.1%). The drug most commonly suspected of causing hypokalaemia was furosemide (6, 3.3%). The drugs most commonly suspected of causing gastrointestinal bleeding were warfarin (3, 1.7%), heparin (3, 1.7%) and diclofenac (3, 1.7%). In general, anti-TB agents constituted the major source of ADRs (45, 25.0%) followed by antivirals (22, 12.2%) and diuretics (19, 10.6%). Table 2 provides a summary of ADRs and causative agents.

**Table 2:** ADRs (N = 119) and implicated drugs (N = 180).

ADR types	n (%)	Drugs implicated in causing ADRs (n)	n (%)
Hepatotoxicity (cholestatic (22), hepatocellular (8), and mixed (5))	35 (29.4)	Isoniazid (21), pyrazinamide (18), atorvastatin (5), efavirenz (5), acetyl salicylic acid (ASA)^#^ (2), clopidogrel (2), propylthiouracil (2), paracetamol (2), phenytoin (2), rifampicin (1), omeprazole (1), ritonavir (1), atazanavir (1)	63 (35.0)
Acute kidney injury (27), and lactic acidosis (1)	28 (23.5)	Tenofovir (9), furosemide (7), enalapril (7), diclofenac (4), acetyl salicylic acid^#^ (4), cimetidine (1), heparin (1), clopidogrel (1), metformin^ (1)	35 (19.4)
Skin reactions involving mucous membrane (pleuritic skin eruption (2), rash (5) and Steven Johnson Syndrome(1))	8 (6.7)	Sulfamethoxazole (2), rifampicin (2), trimethoprim (2), ciprofloxacin* (1), ampicillin (1), isoniazid (1), nevirapine (1), methotrexate (1), cloxacillin (1), phenytoin (1), phenobarbital (1), warfarin (1)	15 (8.3)
Gastrointestinal bleeding (6) and gastritis (1)	7 (5.9)	Warfarin (3), heparin (3), diclofenac (3), clopidogrel (2), acetylsalicylic acid^#^ (2), propylthiouracil (1), glibenclamide (1)	15 (8.3)
Hypokalaemia	7 (5.9)	Furosemide (6), insulin (3), digoxin (1)	10 (5.6)
Hypocalcaemia	6 (5.0)	Furosemide (6)	6 (3.3)
Diarrhoea	5 (4.2)	Lamivudine (3), rifampicin (2), amitriptyline (1)	6 (3.3)
Hypoglycaemia	4 (3.4)	Insulin (4), glibenclamide (1), acetyl salicylic acid (1)	6 (3.3)
Anaemia	4 (3.4)	Sulfamethoxazole (2), trimethoprim (2), methotrexate (1), phenytoin (1)	6 (3.3)
Swelling of tongue (angioedema) (2) and severe dry cough (1)	3 (2.5)	Enalapril (3)	3 (1.7)
Delirium	2 (1.7)	Efavirenz (2)	2 (1.1)
Thrombocytopenia	2 (1.7)	Isoniazid (1), heparin (2)	3 (1.7)
Falls	1 (0.8)	Diazepam (1), thioridazine (1)	2 (1.1)
Osteomalacia	1 (0.8)	Phenobarbital (1), phenytoin (1)	2 (1.1)
Hypotension (orthostatic)	1 (0.8)	Atenolol (1)	1 (0.6)
Syncope	1 (0.8)	Metoprolol (1)	1 (0.6)
Dizziness	1 (0.8)	Quinine (1)	1 (0.6)
Blurred vision	1 (0.8)	Amlodipine (1)	1 (0.6)
Vertigo	1 (0.8)	Metoprolol (1)	1 (0.6)
Vaginal bleeding	1 (0.8)	Medroxyprogesterone acetate (1)	1 (0.6)

^suspected to have caused lactic acidosis,

* suspected to have caused Steven Johnson Syndrome,

^#^high dose (≥325mg) acetyl salicylic acid

### Drugs used

Out of the 4,018 drugs used by the 1,001 patients, 562 (14.0%) were used by patients who experienced ADR-related admissions. One-hundred and eighty of the 562 drugs were implicated in the 119 ADRs. Anti-infectives constituted the major proportion (52.1% versus 40.5%, p = 0.001) followed by cardiovascular system agents (18.9% versus 24.9%, p = 0.013) in both the ADR and non-ADR groups, respectively (Table 3).

**Table 3:** Comparison of drugs used between non-ADR and ADR patient groups.

ATC class name	Non-ADR group n (%)	ADR group n (%)	p-value
Alimentary tract and metabolism	348 (10.1)	57 (10.1)	0.962
Blood and blood forming organs	374 (10.8)	46 (8.1)	0.085
Cardiovascular system	862 (24.9)	106 (18.9)	0.013
Dermatologicals	4 (0.1)	2 (0.4)	0.173
Genito-urinary system and sex hormones	19 (0.5)	2 (0.4)	0.556
Systemic hormonal preparations	96 (2.8)	9 (1.6)	0.113
Anti-infective for systemic use	1398 (40.5)	293 (52.1)	0.001
Antineoplastic and immunomodulating agents	0 (0.0)	1 (0.2)	NA^a^
Musculo-skeletal system	78 (2.3)	9 (1.6)	0.331
Nervous system	188 (5.4)	24 (4.3)	0.273
Antiparasitic products	37 (1.1)	6 (1.1)	0.995
Respiratory system	52 (1.5)	7 (1.2)	0.640
Total	3456 (100.0)	562 (100.0)	

^a^NA = Not applicable, ATC = Anatomical Therapeutic Chemical Classification

### Patient characteristics in univariate analysis

The main socio-demographic difference between the ADR-and non-ADR groups was a difference in urban versus rural residence (P = 0.004), where urban residents were more likely to be admitted with ADRs than rural residents. The mean body mass index (kg/m^2^) was lower in the ADR group, 19.1±2.8 versus 20.1±2.9, p = 0.004. There was no difference between the ADR and non-ADR groups for age, gender, educational status, smoking, alcohol use, khat chewing and herbal use. Patients with a previous ADR history (p<0.001), pre-existing renal diseases (p = 0.003), pre-existing liver diseases (p<0.001), TB taking anti-TB drugs (p<0.001) and HIV/AIDS taking ART (p<0.001) were more likely to be admitted with ADRs than those without these risk factors. Similarly, ADR admission was more likely in patients who had been hospitalised in the preceding 3 months (p = 0.030). Patients in the ADR group had a more comorbidities (4.0±1.4 versus 3.2±1.2, p<0.001) and used more drugs than the non-ADR group (5.5±2.7 versus 3.9±2.1, p<0.001) (Table 4).

**Table 4:** Characteristics of patients experiencing ADR-related admissions and non-ADR-related admissions at Jimma Universality Specialised Hospital.

Risk factors	Non-ADR related admissions	ADR-related admission	p-value
Total patients	N = 898	N = 103	
Age, median (IQR)	40 (28–55)	40 (28–60)	0.490
Gender, female n (%)	404 (45.0)	51 (49.5)	0.382
Body mass index (kg/m^2^), mean ± SD	20.1±2.9	19.1±2.8	0.004
BMI (kg/m^2^), n (%)			
<18.5kg/m^2^	260 (29.0)	44 (42.7)	
>/ = 18.5 kg/m^2^	638 (71.0)	59 (57.3)	0.004
Education, n (%)			
Educated	613 (68.3)	68(66.0)	
Uneducated	285 (31.7)	35(34.0)	0.644
Residence, n (%)			
Urban resident	421 (46.9)	64 (62.1)	
Rural resident	477 (53.1)	39 (37.9)	0.003
Alcohol users, n (%)	218 (24.3)	17 (16.5)	0.080
Khat chewers, n (%)	121 (13.5)	5 (5.9)	0.141
Herbal users, n (%)	73 (8.1)	6 (5.8)	0.710
Previous ADR history, n (%)	12 (1.3)	29 (28.2)	<0.001
eGFR, n (%)			
<60 mL/minute/1.73m^2^	307 (34.2)	27 (26.2)	
≥60 mL/minute/1.73m^2^	376 (41.9)	61 (59.2)	
Unknown	215 (23.9)	15 (14.6)	0.003
Pre-existing renal diseases, n (%)	48 (5.3)	13 (12.6)	0.003
Pre-existing liver diseases, n (%)	70 (7.8)	20 (19.4)	<0.001
Pre-existing heart failure, n (%)	232 (25.8)	27 (26.2)	0.934
HIV/AIDS patients taking ART, n (%)	79 (8.8)	29 (28.2)	<0.001
TB patients taking anti-TB drugs, n (%)	171 (19.0)	36 (35.0)	<0.001
Malignancy of any type, n (%)	40 (4.5)	5 (4.9)	0.853
Cerebrovascular diseases, n (%)	84 (9.4)	4 (3.9)	0.063
Diabetes with complications, n (%)	69 (7.7)	9 (8.7)	0.705
Chronic obstructive pulmonary diseases, n (%)	54 (6.0)	3 (2.9)	0.198
Peptic Ulcer Diseases, n (%)	20 (2.2)	3 (2.9)	0.660
Pre-existing hypertension, n (%)	109 (12.1)	11 (10.7)	0.666
Number of comorbidities, mean ± SD	3.2±1.2	4.1±1.4	<0.001
Number of comorbidities, n (%)			
1–3 comorbidities	563 (62.7)	36 (35.0)	
≥ 4 comorbidities	335 (37.3)	67 (65.0)	<0.001
Number of total medications, mean ± SD	3.9±2.1	5.6±2.7	<0.001
Number of medications, n (%)			
1–5 medications	721 (80.3)	59 (57.3)	
≥ 6 medications	177 (19.7)	44 (42.7)	<0.001
Admission in the preceding 3 months (≥1 admission(s)), n (%)	114 (12.7)	21 (20.4)	0.030

### Factors associated with ADR-related admissions

Variables included in the multivariable binary logistic regression model were those with p<0.25 in the univariate analyses. There was no multicollinearity identified in the included variables. Six risk factors associated with ADR-related hospitalisation were identified in the multivariable binary logistic regression model: BMI < 18.5kg/m^2^, pre-existing renal diseases, pre-existing liver diseases, number of diagnoses ≥4, number of medications ≥6 and previous ADR history (Table 5). The ADR-related admission risk prediction model was found to have an AUROC of 79.0% (95% CI 73.9%-84.1%) (S1 Fig). The model had a sensitivity of 59.2% and specificity of 86.6%, suggesting that it can moderately rule-in patients at risk of ADRs and strongly rule-out those patients not at risk of ADRs.

**Table 5:** Regression model of ADR-related hospitalisation.

Predictors	Crude		Adjusted	
	OR (95%CI)	p-value	OR (95%CI)	p-value
BMI < 18.5kg/m^2^	1.83 (1.21–2.78)	0.004	1.69 (1.10–2.62)	0.047
Pre-existing renal diseases	2.56 (1.34–4.90)	0.005	2.84 (1.38–5.85)	0.004
Pre-existing liver diseases	2.85 (1.65–4.92)	<0.001	2.61 (1.38–4.96)	0.003
Number of comorbidities ≥ 4	3.13 (2.04–4.79)	<0.001	2.09 (1.27–3.44)	0.004
Number of medications ≥ 6	3.04 (1.99–4.64)	<0.001	2.02 (1.26–3.25)	0.004
History of previous ADR	28.94 (14.18–59.05)	<0.001	24.27 (11.29–52.17)	<0.001
Alcohol users	1.62 (0.94–2.79)	0.080	2.45 (1.29–4.65)	0.060
Urban residence	1.86 (1.22–2.83)	0.004	1.50 (0.89–2.52)	0.123
Khat chewers	1.46 (0.88–2.41)	0.141	1.03 (0.58–1.82)	0.932
TB patients taking anti-TB dugs	2.28 (1.47–3.54)	<0.001	1.41 (0.79–2.50)	0.241
HIV/AIDS patients taking ART	4.06 (2.49–6.62)	<0.001	1.33 (0.67–2.65)	0.416
Admission in the preceding 3 months	1.76 (1.05–2.96)	0.030	0.99 (0.51–1.91)	0.965

## Discussion

Identification and reporting of predictors of ADR-related hospitalisation for community-based patients is crucial to develop preventive strategies and responsible care of patients. Medical practitioners may lack awareness of factors predicting ADRs leading to hospitalisation [61, 62]. To overcome this, studies, mainly from developed countries [28, 37], have identified several predictors of ADRs causing hospitalisation. However, Ethiopia is a developing country with different healthcare issues. These issues include a lesser ability to provide healthcare [19], a rising proportion of colliding epidemics of infectious and non-communicable diseases demanding multiple medications with potential of interactions [11, 63], a greater prevalence of HIV/AIDS and TB co-infection [64] with overlapping adverse effects of their medications [6], and a less health-literate population [14]. Additionally, malnutrition and anaemia are more common than in developed countries [65, 66]. Our study, the first in Ethiopia, identified risk factors associated with ADRs, and characterised the reaction types and drugs implicated in admission to the JUSH in Southwest Ethiopia.

We found that 10.3% of admissions were related to ADRs, a prevalence comparable to other studies from South Africa [39] and Argentina [67]. Similarly, a review of studies with a similar design to ours found comparable rates of ADRs in both developed and developing countries [1]. More than half of the ADR-related admissions were due to hepatotoxicity (mainly due to isoniazid and pyrazinamide) and acute kidney injury (mainly due to tenofovir, enalapril and furosemide). Very few studies, only Patel *et al*. [68] from India and Mouton *et al*. [39] from South Africa, have reported comparable proportions of anti-TB-induced hepatotoxicity and tenofovir-induced renal impairment. The higher incidence of hepatotoxicity in the current study could be due to concomitant anti-TB, ART and other medications with overlapping hepatotoxic effects. In addition, there was a substantial number of patients with malnutrition in the current study, and potentially the slow acetylation status of some Ethiopian patients with isoniazid [69] could have exacerbated the hepatotoxic reactions. ADRs commonly responsible for hospitalisation in previous studies included gastrointestinal bleeding [25, 31], electrolyte and metabolic disturbances [8, 28, 36] and cardiovascular disorders [28, 30]. Similar ADRs were reported in the current study, but at lower rates probably due to differences in population, diseases characteristics and drug therapy used.

Most of the predictors of ADRs, such as number of comorbidities [27, 28], number of drugs [28, 29, 36], pre-existing renal failure [31, 70], pre-existing liver diseases [37] and history of previous ADRs [37, 38], were in line with reported findings from both developed and developing countries. HIV/AIDS patients taking ART was identified as an independent predictor that raised the risk of experiencing ADRs in South African studies [8, 39] in 2005 and 2013, respectively. However, this variable was not included in the predictive model in the current study. This is possibly due to the intrinsic relationships between HIV/AIDS and malnutrition, multiple comorbidities and polypharmacy, although multicollinearity was not detected. In addition, the strength of the effect of HIV/AIDS patients taking ART as an independent variable is likely less than that of the other variables possibly because HIV/AIDS patients taking ART would have shown crossover-interaction with ADR-related hospitalisation. Additionally, compared to earlier South African studies, the burden of HIV/AIDS is now markedly reduced due to improvements in health care and greater availability of more tolerable ART. The two South African studies were conducted in communities with a high prevalence of HIV/AIDS, when more toxic antiretroviral drugs were in use. Finally, pharmacogenomic variations and other clinical characteristics of patients may have contributed to this difference [69].

Patients with pre-existing renal diseases were more likely to be hospitalised with ADRs than patients with normal renal function, as has been identified in other studies [71, 72]. About one-third of the patients presented to JUSH with an eGFR <60 mL/min/1.73m^2^ (Table 4), of whom, 18.5% had pre-existing renal diseases. Nearly one-third of patients were underweight (Table 4) and patients with lower BMI were at increased risk of developing ADRs in the current study (Table 5), which is in line with another study [73]. The combined effect of reduced muscle mass (malnutrition) with chronic illnesses may have contributed to a depressed eGFR despite normal serum creatinine levels, and concealed renal insufficiency may impact on the clearance of hydrophilic drugs [74]. In addition, patients in the current study might unintentionally be overdosed based on their weight and/or renal function (using eGFR) that may have led them to develop ADRs. It is therefore important to evaluate renal function to assess the potential therapeutic benefit against the risk of ADRs before initiating renally cleared drugs.

Similar to previous studies [33, 75], patients with pre-existing liver diseases were more likely to develop ADRs. Patients with liver diseases may have multiple comorbidities that require complex medical regimens [76]. Pharmacokinetic and pharmacodynamic changes such as a decreased drug elimination or increased toxic metabolites, alteration in drug distribution or protein binding provide opportunity for adverse reactions [33]. In addition, some drugs (such as anti-TB and ART) commonly used in the sample population are more likely to be associated with hepatotoxicity, in contrast to drugs used in developed countries.

We found that ADR patients were prescribed a higher number of medications compared to non-ADR patients. That means, increasing medical complexity, both number of co-morbidities and number of medications, were associated with an increased risk for ADR-related hospitalisations. This is clearly described in the literature [28, 30, 37].

Patients with a previous ADR history were more likely to be admitted with ADRs which is consistent with other studies [37, 38]. This might be explained by immunological reactions tending to become worse on repeated exposure due to immunologic memory or cross-reaction to alternative drugs, or because no alternative drugs are available more toxic alternatives must be used. Most of the ADRs observed were preventable. Most of the ADRs in the current study occurred due to lack of close review of patients’ previous clinical and medication-related progress. ADR prevention could have been improved with better knowledge of patients’ medical and medication history and associated risk factors.

The ADR-related hospitalisation risk prediction model demonstrated a capacity of 79.0% to discriminate patients who are at risk of ADR-related hospitalisation and those patients who are not. The sensitivity and specificity of the ADR-risk prediction model was 59.2% and 86.6%, respectively, which suggests that it can moderately rule-in patients at risk of ADRs and strongly rule-out those patients not at risk of ADRs. To our knowledge, there is no previously developed similar ADR-risk prediction model in similar study populations aged ≥18 years, especially in low and middle-income countries. Previous models developed by Zopf et al. [37] and Parameswaran et al. [57] showed comparable results (predictive abilities of 80.0% and 70.0%, respectively), but these models were developed in different populations with different clinical characteristics and drug therapy. Our model was further augmented by an ADR preventability assessment using Schumock and Thornton’s preventability assessment criteria, in which the majority of the ADRs were preventable provided these risk factors were reviewed and monitored closely. Although our ADR risk prediction model was not validated in other populations, the majority of the variables identified as independent predictors of ADRs have been described in previous studies [37, 57], suggesting that it is a useful model to assist healthcare practitioners to moderately identify patients at risk for ADRs for implementation of intervention strategies.

### Limitations and strengths

Due to the cross-sectional nature of this study, we were only able to consider the incidence of ADRs at a time, so there is a need for future longitudinal studies to consider the incidence of initial and repeat events, and interventional strategies to identify root causes and reduce ADR-related burdens. The self-reported responses for previous ADR history, for instance, may be limited by recall bias and could have influenced the identified predictors of ADRs, especially in patients taking multiple medications for chronic illnesses. Some questions in the Naranjo causality assessment tool were designed for controlled clinical trials; they were not feasible nor ethical for clinical practice, such as observing effects on giving placebo. Commonly used over-the-counter medicines, contraceptives, topical agents, and herbal remedies were not typically recorded in drug histories, which may have resulted in underestimation of the rate of ADR-related admissions. The results of this study should be extrapolated to other countries with caution, as the study findings depend on the patient characteristics, disease distribution, healthcare infrastructure, detection methods and definitions of ADRs adopted.

The strength of our study is the prospective identification of ADRs immediately upon admission, allowing for accurate evaluation of the clinical presentation and laboratory parameters. We used a three-step process in ADR assessment, evaluating individual patient’s clinical and laboratory parameters, causality assessment using the Naranjo algorithm followed by consensus review with a senior supervising internist, to help mitigate the subjectivity associated with interpretation of some ADRs. Our study is the first to identify independent predictors of ADR-related hospitalisation in Ethiopian patients. Our yearlong sampling avoided the bias associated with anti-infective use pattern due to seasonal variation. Our study was conducted in a teaching and referral hospital serving a population of 15 million; therefore, our results allow extrapolation to other settings in the southwest of Ethiopia.

## Conclusions

ADRs were a common cause of hospitalisation in adults admitted to medical wards of the JUSH. The majority of ADRs were preventable, highlighting the need for close monitoring and review of patients with lower BMI, previous ADR history, pre-existing renal and liver diseases, multiple comorbidities and medications. The ADR-related hospitalisation risk prediction model demonstrated some ability to identify patients at higher risk for ADRs, and clearly identify patients at lower risk of ADRs. ADR predictors should be integrated into clinical pathways and pharmacovigilance systems. However, validation and refinement of the model is necessary prior to its implementation in routine clinical practice. The prevention of incident ADR may be of paramount importance, as previous ADR was a strong predictor of subsequent events in this patient population. Assessment of ADR causality and effective use of a pharmacovigilance system to monitor drug response in patients should be considered at ambulatory care units of all health care levels to minimise the burden of admissions related to ADRs by targeting the occurrence of preventable reactions.

## Supporting information

S1 Fig Area under the receiver operator curve (AUROC) showing ADR-risk prediction capacity of the model. (TIF)

S1 Table Data collection tool. (DOCX)

S2 Table Dataset. (ZIP)
