# Supplementary figures and images for: Predictors of adverse drug reaction-related hospitalisation in Southwest Ethiopia: A prospective cross-sectional study
Source: PLoS One. 2017 Oct 16;12(10):e0186631. doi: 10.1371/journal.pone.0186631 (PMC5643118; doi:10.1371/journal.pone.0186631)

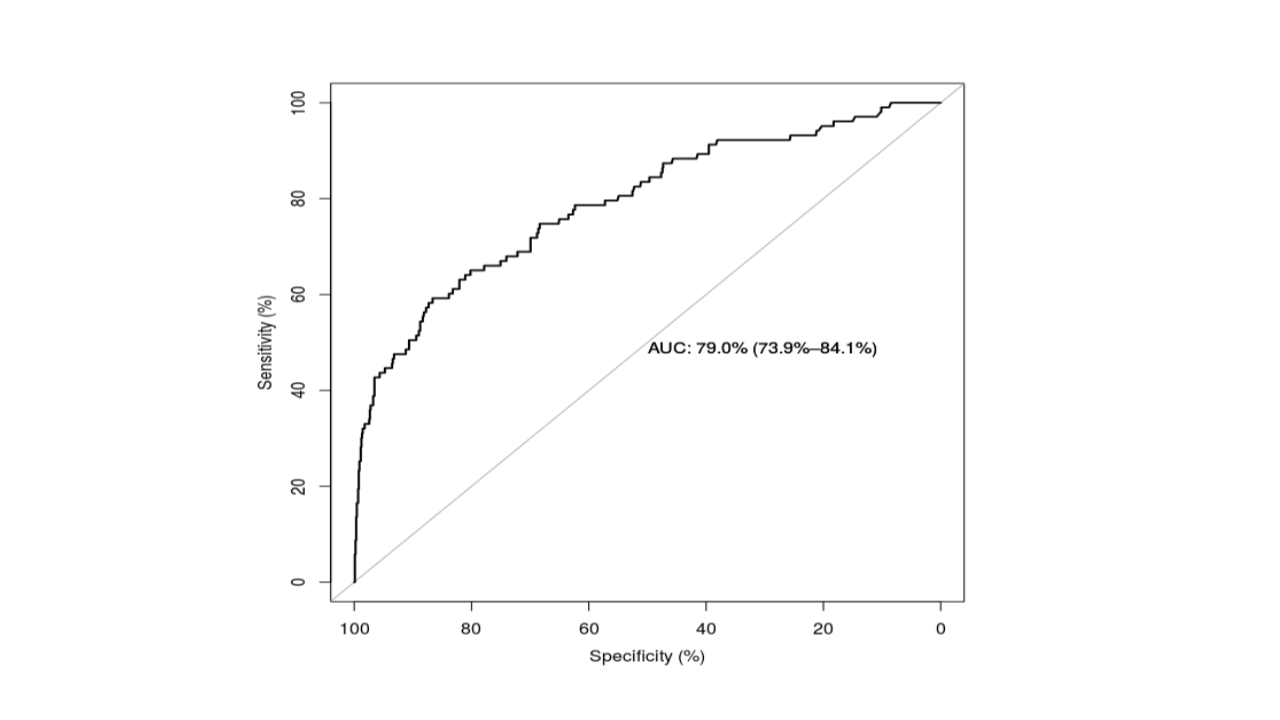

Supplement: S1 Fig — (TIF) [file pone.0186631.s001.tif]
