# Supplementary material for: Predictors of adverse drug reaction-related hospitalisation in Southwest Ethiopia: A prospective cross-sectional study
Source: PLoS One. 2017 Oct 16;12(10):e0186631. doi: 10.1371/journal.pone.0186631 (PMC5643118; doi:10.1371/journal.pone.0186631)
Supplement: S1 Table — (DOCX) [file pone.0186631.s002.docx]

# **S1 Table: Data Collection Tool**

**Section I: Socio-demographic and life style characteristics**

Patient card number_________________________Referral source___________________

1. Admission date _____________________(dd/mm/yyyy)
2. Discharge date______________________(dd/mm/yyyy), Fatality: A. Yes B. No
3. Age_______________
4. Gender: a) Male b) Female
5. Weight(kg) _____________
6. Height(m)_____________
7. Educational status:
8. Illiterate
9. Primary school
10. Secondary school
11. College and above
12. Marital status:
13. Single
14. Married
15. Widowed
16. Divorced
17. Job/ Occupation:
18. Farmer
19. Merchant
20. Government employee
21. Non-government employee
22. Private employee
23. Private technical works (e.g. carpenter )
24. Retired
25. Student
26. Others, specify _______________
27. Residence/Place of living:
28. Rural village
29. Urban (city or town)
30. Living status
31. Living with immediate family
32. Living with extended family
33. Living alone
34. Other, specify________________
35. How often do you consume alcohol (Beer, Wine, “Tella”,”Tejji”, “Katikalla”, etc)?
36. Usually( daily )
37. Sometimes (2-4 times per week)
38. Rarely (≤1 times per week)
39. Not at all
40. Unknown
41. Do you smoke cigarette?
42. Yes
43. No
44. Unknown

If YES, how many cigarette per day ___________________________

1. Khat chewing habit:
2. Regular (≥4 times per week)
3. Sometimes (2-3 times per week)
4. Rarely (≤1 times per week)
5. Not chew khat
6. Unknown
7. Do you use herbal/alternative medicine in your home or among practitioners?
8. Yes
9. No
10. Unknown

If YES, the name of the herb/product___________________________________

**Section II: Patient’s Cognition, daily Activity and Medication adherence (Interviews)**

1. Abbreviated Mental Test (AMT): Used for cognitive status assessment

| S. No | Questions | Yes=1 | No =0 |
| --- | --- | --- | --- |
|  | How old are you? |  |  |
|  | What is the time (nearest hour)? |  |  |
|  | Address for recall at the end of test – this should be repeated by the patient, e.g. Ferenji Arada |  |  |
|  | What year is it? |  |  |
|  | What is the name of this place? |  |  |
|  | Can the patient recognise two relevant persons (e.g. Nurse/Doctor) |  |  |
|  | What was the date of your birth? |  |  |
|  | When was Ethio-Eritrean War? |  |  |
|  | Who is the present Prime Minister? |  |  |
|  | Count down from 20 to 1 (no errors, no cues) |  |  |
| **Total Correct sum** | | ______/10 | |

NB: A score of less than 7 suggests cognitive impairment.

1. Barthel Index (BI) of Activities of Daily Living

|  | **Variables** | **With help** | **Independent** |
| --- | --- | --- | --- |
| 1 | Feeding (if food needs to be cut up) | 5 | 10 |
| 2 | Moving from wheelchair to bed and return (includes sitting up in bed) | 5-10 | 15 |
| 3 | Personal toilet (wash face, comb hair, shave, clean teeth) | 0 | 5 |
| 4 | Getting on and off toilet (handling clothes,wipe,flush) | 5 | 10 |
| 5 | Bathing self | 0 | 5 |
| 6 | Walking on level surface(or if unable to walk, propel wheelchair)  *score only if unable to walk | 10  0* | 15  5* |
| 7 | Ascend and descend stairs | 5 | 10 |
| 8 | Dressing(includes tying shoes, fastening fasteners) | 5 | 10 |
| 9 | Controlling bowels | 5 | 10 |
| 10 | Controlling bladder | 5 | 10 |
| **Total Correct sum** | | _________/100 | |

**Notice:** A patient scoring 100 BI is continent, feds himself, dresses himself, gets up out bed and chairs, bathes himself, walks at least a block, and can ascend and descend stairs.

1. Morisky 8-Item Medication Adherence scale

| **No.** | **Morisky 8-Item Medication Adherence Questionnaire** | **Answer** |
| --- | --- | --- |
| 1 | Do you sometimes forget to take your medicine? |  Yes  No |
| 2 | People sometimes miss taking their medicines for reasons other than forgetting. Thinking over the past 2 weeks, were there any days when you did not take your medicine? |  Yes  No |
| 3 | Have you ever cut back or stopped taking your medicine without telling your doctor because you felt worse when you took it? |  Yes  No |
| 4 | When you travel or leave home, do you sometimes forget to bring along your medicine? |  Yes  No |
| 5 | Did you take all your medicines yesterday? |  Yes  No |
| 6 | When you feel like your symptoms are under control, do you sometimes stop taking your medicine? |  Yes  No |
| 7 | Taking medicine every day is a real inconvenience for some people. Do you ever feel hassled about sticking to your treatment plan? |  Yes  No |
| 8 | How often do you have difﬁculty remembering to take all your medicine? |  Never/rarely   Once in a while   Sometimes   Usually   All the time |
| **Total Correct sum** | | ______/8 |

**Notice:** Never/rarely= 0; Once in a while/ Sometimes/Usually/All the time = 1 and Yes=1 and No=0

**Section III: Medical and medication related data (Medical Record Review)**

1. Chief compliant for current admission is : _____________________________________________________________________
2. Pertinent history of present illness:

______________________________________________________________________________________________________________________________________________________Current physician diagnosis at admission

_______________________________________________________________________

1. Was the patient following up for chronic care at ambulatory care clinic?

a) Yes b) No c) unknown

1. Was the patient admitted in the last 3 months?

a) Yes b) No c) unknown

If YES, reasons for admission______________________________________________ and Number of admission________________

1. Does the patient have documented or reported drug allergy/hypersensitivity history, e.g. to Penicillin, Aspirin?

a) Yes b) No c) unknown

If YES, for which drug/agent_______________________________________________

1. Does the patient have documented or reported ADR history?

a) Yes b) No c) unknown

If YES, for which drug/agent ______________________________________________

1. The Charlson Co-Morbidity Index

| **The Charlson Co-Morbidity Index*** | **Yes** | **No** | **Classic score** | **Updated** |
| --- | --- | --- | --- | --- |
| AIDS |  |  | +6 | +4 |
| Cerebrovascular disease (excluding hemiplegia) |  |  | +1 | +0 |
| Chronic respiratory disease (e.g. asthma, COPD, bronchitis) |  |  | +1 | +1 |
| Congestive heart failure |  |  | +1 | +2 |
| Connective tissue disease (e.g. SLE, rheumatoid arthritis, scleroderma, Sjogren’s disease, osteoarthritis, etc.) |  |  | +1 | +1 |
| Dementia |  |  | +1 | +2 |
| Hemiplegia or paraplegia |  |  | +2 | +2 |
| Any malignancy including Leukaemia and Lymphoma |  |  | +2 | +2 |
| Myocardial infarction |  |  | +1 | +0 |
| Peripheral vascular disease |  |  | +1 | 0 + 0 |
| Peptic ulcer disease |  |  | +1 | + 0 |
| Diabetes mellitus without end organ damage |  |  | +1 | + 0 |
| Diabetes mellitus with end organ damage |  |  | +2 | +1 |
| Mild liver disease (e.g. cirrhosis without portal HT, chronic hepatitis) |  |  | +1 | +2 |
| Moderate or severe liver disease (e.g. cirrhosis with portal HT +/- variceal bleeding) |  |  | +3 | +4 |
| Renal diseases |  |  | +2 | +1 |
| Metastatic solid tumour |  |  | +6 | +6 |
| **Total score:** | | |  |  |
| **Age of patient**  1. Age <40 years: 0 points  2. Age 41‐50 years: 1 points  3. Age 51‐60 years: 2 points  4. Age 61‐70 years: 3 points  5. Age 71‐80 years: 4 points  6. Age 81‐89 years: 5 points | | | +0  +1  +2  +3  +4  +5 | +0  +1  +2  +3  +4  +5 |
| **Total score, including age factor** | | |  |  |
| ***Note:** The following comorbid conditions are mutually exclusive: diabetes with chronic complications and diabetes without chronic complications; mild and moderate/sever liver disease; and any malignancy and metastatic solid tumour | | | | |

1. Pertinent Laboratory/Biochemical investigation results that could be helpful for ADR diagnosis (write the laboratory investigation result in-front of respective parameters if it was performed as per the patient clinical indication )

| Laboratory parameters | Laboratory investigations vs normal reference values | | | | |
| --- | --- | --- | --- | --- | --- |
|  | At Admission (if any between 0 to 48 hrs of admission) and baseline values | | Reference values (Bases on Koda-Kimble, et.al. Applied therapeutics book) | | |
| **Renal function test** |  |  | |  |  |
| 1. Scr |  | | 0.6-1.2mg/dl | | |
| 1. BUN |  | | 8-18 mg/dl | | |
| 1. eGFR |  | | 75-125ml/min | | |
| **Liver function test** |  |  | |  |  |
| 1. AST |  | | 0-35 IU/L | | |
| 1. ALT |  | | 0-35IU/L | | |
| 1. ALP |  | | 30 – 120 U/L | | |
| 1. Bilirubin-total |  | | 0.1–1.0 mg/dL | | |
| 1. Bilirubin-direct |  | | 0-0.2mg/dL | | |
| 1. Serum albumin |  | | 3.5 – 5 g/dL | | |
| **Complete Blood Count(CBC)** |  |  | |  |  |
| 1. WBC count |  | | 3.2-9.8x10^3^cell/mm^3^ | | |
| 1. RBC count |  | | 4.2-5.9x10^6^ for M&3.5-5x10^6^ cells/mm^3^ forF | | |
| 1. Neutrophils |  | | 54%-62% | | |
| 1. Bands |  | | 3%-5% | | |
| 1. Lymphocytes |  | | 25%-33% | | |
| 1. Monocytes |  | | 3%-7% | | |
| 1. Eosinophils |  | | 1%-3% | | |
| 1. Basophils |  | | <1% | | |
| 1. Hgb |  | | 14-18g/dL for M &12-16 g/dL for females | | |
| 1. HCT |  | | 39-49% for M &33-43% for F | | |
| 1. MCV |  | | 76 to 100 *μ*m^3^ | | |
| 1. MCH |  | | 27 to 33pg | | |
| 1. MCHC |  | | 33- 37g/dL | | |
| 1. PLT |  | | 130-400x10^3^/ mm^3^ | | |
| **Coagulation Test** |  |  | |  |  |
| 1. INR |  | | 2.0-3.0 for AF/DVT/VHD | | |
| 1. aPTT |  | | 35 to 45 to seconds | | |
| **Lipid panel** |  |  | |  |  |
| 1. TG |  | | <160 mg/dL | | |
| 1. Total Cholest |  | | <200 mg/dL | | |
| 1. LDL |  | | 70–160 mg/dL | | |
| 1. HDL |  | | >45 mg/dL | | |
| **Cardiac function** |  |  | |  |  |
| 1. Troponin I |  | | <0.03 ng/mL | | |
| 1. CK-MB |  | | 0–12 units/L | | |
| **Electrolyte test** |  |  | |  |  |
| 1. Na+ |  | | 135 - 145 mEq/L | | |
| 1. K+ |  | | 3.5 – 5.0 mEq/L | | |
| 1. Ca+ |  | | 8.8 – 10.2 mg/dl | | |
| **Glycaemic level** |  |  | |  |  |
| 1. FPG |  | | 70 – 110 mg/dl | | |
| 1. RPG |  | | 140-180mg/dl | | |
| **Vital signs** |  |  |  | | |
| 1. Temperature |  | |  | | |
| 1. BP |  | |  | | |
| 1. PR |  | |  | | |
| 1. RR |  | |  | | |
| Ultrasonography interpretation |  | | Based on patient condition | | |
| ECHO interpretation |  | | Based on patient condition | | |
| EKG interpretation |  | | Based on patient condition | | |
| Others |  | |  | | |

1. List of medications (including OTC medications and contraceptive pills) used **prior to the current hospital admission**

| Name of the prescription only drug  Yes  No | Dose | Frequency | Date started | Date stopped | Routes | Remark |
| --- | --- | --- | --- | --- | --- | --- |
|  |  |  |  |  |  |  |
|  |  |  |  |  |  |  |
|  |  |  |  |  |  |  |
|  |  |  |  |  |  |  |
|  |  |  |  |  |  |  |
|  |  |  |  |  |  |  |
|  |  |  |  |  |  |  |
|  |  |  |  |  |  |  |
|  |  |  |  |  |  |  |
| Name of Contraceptives  Yes  No | Dose | Frequency | Date started | Date stopped | Routes | Remark |
|  |  |  |  |  |  |  |
|  |  |  |  |  |  |  |
| Name of OTC drugs  Yes  No | Dose | Frequency | Date started | Date stopped | Routes | Remark |
|  |  |  |  |  |  |  |
|  |  |  |  |  |  |  |
|  |  |  |  |  |  |  |

**Section IV: Summary of ADR Case note**

1. Describe the suspected adverse reactions based on the above available patient data.

1.

2.

3.

4.

1. What are the pertinent clinical and laboratory abnormalities for suspected corresponding drug(s)?

| Pertinent suspected drug related reactions (after interviewing of patient and reviewing of patient medical record) i.e. before causality assessment: **Clinical data** | Pertinent **biochemical /laboratory** abnormality detected | Corresponding suspected list of drug(s) patient taking at admission |
| --- | --- | --- |
|  |  |  |
|  |  |  |
|  |  |  |
|  |  |  |

1. Suspected drug outcomes
2. Drug stopped
3. Drug withheld
4. Drug continued
5. Drug substituted
6. Dose reduced
7. Antidote or counteracting agent administered
8. Unknown
9. ADR outcome:
10. Fatal
11. Not yet recovered
12. Recovered
13. Unknown

**Section V: ADR Causality, Severity, Type and Preventability assessment**

1. Causality assessment for each of the suspected drug on Q28 above

| **Question** | **Yes** | **No** | **Do Not Know** | **Scores for suspected**  **drug(s) in Q31** | | | |
| --- | --- | --- | --- | --- | --- | --- | --- |
|  |  |  |  | **(1)** | **(2)** | **(3)** | **(4)** |
| 1. Are there previous conclusive reports on this reaction? | +1 | 0 | 0 |  |  |  |  |
| 1. Did the adverse reaction appear after the suspected drug was administered? | +2 | -1 | 0 |  |  |  |  |
| 1. Did the adverse reaction improve when the drug was discontinued or a specific antagonist was administered? | +1 | 0 | 0 |  |  |  |  |
| 1. Did the adverse reaction reappear when the drug was readministered? | +2 | -1 | 0 |  |  |  |  |
| 1. Are there alternative causes that could on their own have caused the reaction? | -1 | +2 | 0 |  |  |  |  |
| 1. Did the reaction reappear when a placebo was given? | -1 | +1 | 0 |  |  |  |  |
| 1. Was the drug detected in blood or other fluids in concentrations known to be toxic? | +1 | 0 | 0 |  |  |  |  |
| 1. Was the reaction more severe when the dose was increased or less severe when the dose was decreased? | +1 | 0 | 0 |  |  |  |  |
| 1. Did the patient have a similar reaction to the same or similar drugs in any previous exposure? | +1 | 0 | 0 |  |  |  |  |
| 1. Was the adverse reaction confirmed by any objective evidence? | +1 | 0 | 0 |  |  |  |  |
| **Total Score:** |  |  |  |  |  |  |  |

1. List of drug(s) implicated in causing ADR

| List of drug(s) | List of corresponding ADRs |
| --- | --- |
|  |  |
|  |  |
|  |  |
|  |  |

1. Type of ADR based on Rawlins classification (Tick “√” in the most appropriate box taking into consideration of ADR listed in Q33)

| ADR type | Reaction (1) | Reaction (2) | Reaction (3) | Reaction (4) |
| --- | --- | --- | --- | --- |
| 1. Type A (Dose dependent, pharmacological and predictable) |  |  |  |  |
| 1. Type B (Dose independent, bizarre and non-predictable) |  |  |  |  |

1. Is there clinically significant interactions between or among drugs used by the patient at admission? This question should be answered by checking interactions in MICROMEDEX drug interaction checker and based on existing evidences.

a) Yes b) No c) Unknown

If YES, the severity level of interaction is:

1. **Contraindicated** (Avoid combination)-means the drugs are contraindicated for concurrent use
2. **Major** (Consider therapy modification) -means the interaction may be life threatening and/or require medical intervention to minimize or prevent serious adverse events
3. **Moderate** (Monitor therapy)-means the interaction may result in exacerbation of the patient’s condition and/or require an alteration in therapy
4. **Minor** (No action is needed) – means the interaction would have limited clinical effects. May include an increase in the frequency or severity of the side effects, but generally it would not require a major alteration in therapy
5. **Unknown** (No known interaction)-the interaction is unknown
6. Severity of ADR based on modified Hartwig and Siegel scale (Tick √ in the most appropriate level for ADR listed in Q33)

| Severity scale | Severity Level | Description of the above identified reaction(s) | Reaction (1) | Reaction (2) | Reaction (3) | Reaction (4) |
| --- | --- | --- | --- | --- | --- | --- |
| 1. Mild | Level 1 | The ADR requires no change in treatment with the suspected drug.  Yes  No |  |  |  |  |
|  | Level 2 | The ADR requires that the suspected drug be withheld, discontinued or otherwise changed.  Yes  No |  |  |  |  |
| 1. Moderate | Level 3 | The ADR requires that the suspected drug be withheld, discontinued or otherwise changed, and/ or an antidote or other treatment is required. There is no increase in lenght of stay.   Yes  No |  |  |  |  |
|  | Level 4 (a) | Any level 3 ADR that increases lenght of stay by at least one day.   Yes  No |  |  |  |  |
|  | Level 4 (b) | The ADR is the reason for admission.  Yes  No |  |  |  |  |
| 1. Severe | Level 5 | Any level 4 ADR that requires intensive medical care.   Yes  No |  |  |  |  |
|  | Level 6 | The ADR causes parmanent harm to the patient.  Yes  No |  |  |  |  |
|  | Level 7(a) | The ADR directly leads to the death of the patient.  Yes  No |  |  |  |  |
|  | Level 7(b) | The ADR indirectly leads to the death of the patient. Yes  No |  |  |  |  |

1. Preventability of ADR based on Schumock and Thornton scale (Tick √ in the most appropriate preventability scale bases on the ADR listed in Q 33)

Notice: Answering “yes” to one or more of the questions in section “a” implies that an ADR is DEFINITELY preventable and If answers are all negative to section “a”, then proceed to Section “b”. Answering “yes” to one or more of the questions in section “b” implies that an ADR is PROBABLY preventable and if the answers are all negative to section “b”, then proceed to Section “c”. In Section “c” the ADR is NOT preventable

| Preventability scale | Description of each scale | Reaction (1) | Reaction (2) | Reaction (3) | Reaction (4) |
| --- | --- | --- | --- | --- | --- |
| 1. Definitely Preventable | 1. Was there a history of allergy or previous reactions to the drug?  Yes  No 2. Was the drug involved inappropriate for the patient’s clinical condition?  Yes  No 3. Was the dose, route or frequency of administration inappropriate for the patient’s age, weight or disease state?    Yes  No   1. Was a toxic serum drug concentration (or laboratory monitoring test) documented?  Yes  No 2. Was there a known treatment for the adverse drug reaction?    Yes  No |  |  |  |  |
| 1. Probably Preventable | 1. Was required therapeutic drug monitoring or other necessary laboratory tests not performed?  Yes  No 2. Was a drug interaction involved in the ADR?  Yes  No 3. Was poor compliance involved in the ADR?  Yes  No 4. Were preventative measures not prescribed or administered to the patient?  Yes  No |  |  |  |  |
| 1. Not preventable | If all above criteria not fulfilled |  |  |  |  |
